# Effect of socioeconomic status on stage at diagnosis of lung cancer in a hospital‐based multicenter retrospective clinical epidemiological study in China, 2005–2014

**DOI:** 10.1002/cam4.1170

**Published:** 2017-09-21

**Authors:** Yuanqiu Li, Jufang Shi, Shicheng Yu, Le Wang, Jianjun Liu, Jiansong Ren, Shugeng Gao, Zhouguang Hui, Junling Li, Ning Wu, Boyan Yang, Shangmei Liu, Mingfang Qin, Debin Wang, Xianzhen Liao, Xiaojing Xing, Lingbin Du, Li Yang, Yuqin Liu, Yongzhen Zhang, Kai Zhang, Youlin Qiao, Jie He, Min Dai, Hongyan Yao, the Health Economic Evaluation Working Group, Cancer Screening Program in Urban China (CanSPUC)

**Affiliations:** ^1^ Office of Epidemiology Chinese Center for Disease Control and Prevention Beijing China; ^2^ Program Office for Cancer Screening in Urban China National Cancer Center/Cancer Hospital Chinese Academy of Medical Sciences and Peking Union Medical College Beijing China; ^3^ Chinese Center for Disease Control and Prevention Beijing China; ^4^ Department of Thoracic Surgery National Cancer Center/Cancer Hospital Chinese Academy of Medical Sciences and Peking Union Medical College Beijing China; ^5^ Department of Radiation Oncology National Cancer Center/Cancer Hospital Chinese Academy of Medical Sciences and Peking Union Medical College Beijing China; ^6^ Department of Medical Oncology National Cancer Center/Cancer Hospital Chinese Academy of Medical Sciences and Peking Union Medical College Beijing China; ^7^ Department of Diagnostic Radiology National Cancer Center/Cancer Hospital Chinese Academy of Medical Sciences and Peking Union Medical College Beijing China; ^8^ Department of General Medicine National Cancer Center/Cancer Hospital Chinese Academy of Medical Sciences and Peking Union Medical College Beijing China; ^9^ Department of Pathology National Cancer Center/Cancer Hospital Chinese Academy of Medical Sciences and Peking Union Medical College Beijing China; ^10^ Division for Chronic Non‐communicable Disease Prevention and Control Yunnan Center for Disease Control and Prevention Kunming China; ^11^ School of Health Services Management Anhui Medical University Hefei China; ^12^ Hunan Office for Cancer Control and Research Hunan Cancer Hospital Changsha China; ^13^ Liaoning Office for Cancer Control and Research Liaoning Cancer Hospital & Institute Shenyang China; ^14^ Zhejiang Office for Cancer Control and Research Zhejiang Cancer Hospital Hangzhou China; ^15^ School of Public Health Guangxi Medical University Nanning China; ^16^ Cancer Epidemiology Research Center Gansu Provincial Cancer Hospital Lanzhou China; ^17^ Department of Epidemiology Shanxi Provincial Cancer Hospital Taiyuan China; ^18^ Cancer Department of Physical Examination National Cancer Center/Cancer Hospital Chinese Academy of Medical Sciences and Peking Union Medical College Beijing China; ^19^ Department of Epidemiology National Cancer Center/Cancer Hospital Chinese Academy of Medical Sciences and Peking Union Medical College Beijing China

**Keywords:** Cancer stage, epidemiology, lung cancer, social class, socioeconomic status

## Abstract

There is inconsistent evidence of associations between socioeconomic status (SES) and lung cancer stage in non‐Chinese populations up to now. We set out to determine how SES affects stage at diagnosis at both individual and area levels, from a hospital‐based multicenter 10‐year (2005–2014) retrospective clinical epidemiological study of 7184 primary lung cancer patients in mainland China. Individual‐level SES data were measured based on two indicators from case report forms of the study: an individual's education and occupation. Seven census indicator variables were used as surrogates for the area‐level SES with principal component analysis (PCA). Multivariate analysis was undertaken using binary logistic regressions and multinomial logit model to describe the association and explore the effect across tertiles on stage after adjusting for demographic variables. There was a significant stepwise gradient of effect across different stages in the highest tertile of area‐level SES, comparing with the lowest tertile of area‐level SES (ORs, 0.77, 0.67, and 0.29 for stage II, III, and IV). Patients with higher education were less likely to have stage IV lung cancer, comparing with the illiterate group (ORs, 0.52, 0.63, 0.71, 0.64 for primary school, middle school, high school, college degree or above subgroup, respectively). Findings suggest that the most socioeconomically deprived areas may be associated with a higher risk of advanced‐stage lung cancer, and increasing educational level may be correlated with a lower risk to be diagnosed at advanced stage in both men and women.

## Introduction

Lung cancer is the leading cause of cancer death and the most common incident cancer in China. Strong social gradients in lung cancer incidence have been observed in China, with significantly higher age‐standardized incidence rate in urban areas than in rural areas in 2015 (445.0 vs. 288.3 per 100,000) 1. In China, highly unequal distribution of insurance benefits still exists under the current social health insurance programs, especially for the vulnerable groups such as children, women, low‐income, and rural population 2, 3. The possible underlying causes of the different cancer outcomes among social groups include health care provision according to socioeconomic areas 4, 5, 6, health‐related behaviors of individuals 7, 8, and environmental or occupational exposures across socioeconomic groups 9, 10, 11. Up to now, there is inconsistent evidence of the association between socioeconomic status (SES) and lung cancer stage from the current studies in non‐Chinese populations. One Scottish study found that rates of earlystage cancer were higher in more deprived patients than less deprived 12. Similarly, a US study showed that college graduates were more likely to be diagnosed with advanced stage at diagnosis compared with those without a college degree 13. However, findings from a recent systematic review and meta‐analysis have not shown the socioeconomic inequalities in late stage at diagnosis in the most studies, compared with the least, deprived group 14, most of included studies (5/7) were from the UK and USA.

China is experiencing urbanization at an unprecedented rate over the last two decades 15. Moreover, the proceeding ambitious healthcare reforms aim to achieve equitable access to basic health services, and to build a safe, fair, and effective healthcare system for both urban and rural residents. The socioeconomic measurements have proved useful to monitor health inequalities and can provide fundamental implications for prevention initiatives and resource allocation 16, 17, 18. To date, the role of SES, either at individual or area level, in shaping lung cancer risk has only been examined from outside of China 9, 12, 19, 20, 21. Since each study has used different variables and different approaches to estimate individual or neighborhood socioeconomic conditions, the accumulated evidence is difficult to assess systematically in China.

Albeit the best way to measure the extent to which the SES has influenced the health inequalities is to link electronic medical records from hospitals and census data from the bureau of statistics for the target population. For now, the electronic health record systems and electronic medical systems in China are still in their infancy, and the information is not available for researchers. In this study, we set out to determine how SES affects stage at diagnosis at both individual and area levels, from a hospital‐based multicenter 10‐year (2005–2014) retrospective clinical epidemiological study for primary lung cancer in mainland China. Furthermore, the paper outlines a reproducible approach to the individual and area deprivation index based on readily available data, and explores the joint effects of different levels of SES, gender, age, smoking, and other demographic characteristics in relation to stages of primary lung cancer.

## Materials and Methods

### Study design

This study was a hospital‐based multicenter 10‐year (2005–2014) retrospective clinical epidemiological study of primary lung cancer cases via medical chart review.

### Hospital selection, case sampling, and data collection

As part of the Cancer Screening Program in Urban China (CanSPUC) supported by the central government 22, the current survey was conducted in eight tertiary hospitals in eight provinces across China between March 2015 and August 2016. According to the traditional administrative district definition by the National Bureau of Statistics, China is stratified into seven geographic regions (North, Northeast, Central, South, East, Northwest, and Southwest), of which population urbanization and economic development level would vary widely. However, the national representativeness was taken carefully into account when selecting the hospitals. The sampling framework consisted of the highest level cancer hospital in each region (eight hospitals in seven regions). Finally eight tertiary hospitals from seven regions were included in this study using convenience sampling, which were Shanxi Provincial Cancer Hospital (in Shanxi Province, north China), Liaoning Cancer Hospital (in Liaoning Province, northeast China), Zhejiang Cancer Hospital (in Zhejiang Province, east China), Anhui Cancer Hospital (in Anhui Province, east China), Hunan Cancer Hospital (in Hunan Province, central China), Guangxi Cancer Hospital (in Guangxi Autonomous Region, south China), Yunnan Cancer Hospital (in Yunnan Province, southwest China), and Gansu Provincial Cancer Hospital (in Gansu Province, northwest China).

The medical records of primary lung cancer patients diagnosed between 2005 and 2014 were collected by well‐trained health professionals over a period of 2 years from 2015 to 2016. It can be summarized as follows.

*Step 1: Using random assignment to the month*. One of the months every year in each hospital was randomly selected to review the entire cases except for January and February, in which the whole Chinese people celebrated their Spring Festival and fly home to spend the holiday with family. There were much fewer patients during the time period and might be a potential confounding in valid outcomes comparison.

*Step 2: Specify inclusion criteria for cases*. The cases contained in medical records database should be older than 18 years and have completed information relating to key demographic and lifestyle factors, diagnostic information (pathologic TNM stage or clinical TNM stage), surgery approaches, use of radiotherapy and chemotherapy, molecular targeted therapy, and pathologic characteristics for lung cancer. Those who were only diagnosed with lung cancer or underwent surgery followed up after surgery without any treatment in the hospital were excluded from our study.

*Step 3: Conduct a thorough review*. The medical records were reviewed in each local hospital by local clerks who had been trained systematically. The clerks started to choose in a forward manner from the first day of selected month. As soon as he/she extracted a hundred medical reports in this month, he/she would turn to other randomized month in the next year. The clerks would indicate reasons for inclusion or exclusion in a special designed table to verify the accuracy of recorded information at the same time. The contents of the case report form (CRF) were designed into an initial questionnaire by experts from cancer epidemiology, pathology, imaging diagnosis, thoracic surgery, medical oncology, radiation oncology, and general medicine, and after a presurvey, revised repeatedly into a formal questionnaire eventually. It was used to extract the information of medical reports as described above.

*Step 4: Recode the raw variables*. According to the designed CRF, the raw variables were encoded for analysis. All the variables were double‐entered from the paper or electronic medical record to computer‐based database (EpiData 3.1) by two local well‐trained clerks, and then were sent to National Office of CanSPUC for the data check. Patients were assigned stage based on pathologic TNM when available and clinical TNM otherwise. Staging was categorized into seven groups based on the seventh edition of American Joint Committee on Cancer (AJCC) tumor‐node‐metastasis (TNM) staging system for lung cancer (I, IIA, IIB, IIIA, IIIB, IV, and unknown or not applicable stage). Advanced stage refers to stage IIIB‐IV, with stage I‐IIIA classifying nonadvanced‐stage cancer. The stage was independently classified and checked by experienced clinicians blinded to the patients’ deprivation status.

In order to be consistent with the other research findings in the future based on the study, we included extensive/localized stage small‐cell lung cancer cases without clinical stage I‐IV information in the descriptive analysis. When conducting multivariate analysis, we excluded 171 small‐cell lung cancer cases due to unknown or not applicable stage. As a result, the final study population consisted of 7184 individuals (5262 men and 1922 women). A total of 7013 cases (5119 men and 1894 women) were used for exploring the association between SES and stage at diagnosis.

The study protocol was approved by the Institutional Review Board of the Cancer Hospital of Chinese Academy of Medical Sciences.

### Measurement of individual‐level and area‐level SES characteristics

Individual‐level SES data were obtained and measured based on two indicators from CRF of the retrospective survey: an individual's educational level and occupational rank, based on Hollingshead's personal rating of people's relative social standing in New Haven, CT, in the early 1960s 23. Education represents knowledge and skills; occupation captures material and social resources and assets 9. Education was categorized to five levels: illiterate, primary school, middle school, high school, and college degree or above. With regard to occupation, both European and United States conceptualized occupations as a social relationship based on a graded hierarchy of occupations ranked according to skill 23. Although such definition of occupation was the best representation of SES, the raw data were not available in China. Given the farmers or rural migrant workers as a huge and special population in China, occupation in our study was classified into two categories: farmer/migrant workers; nonfarmer/nonmigrant workers.

The CRF does not collect area‐level SES characteristics. Therefore, we used the census data to reflect the influence of area‐based SES. We attempted to recreate some indices because some census indicator variables of SES (such as percent older than 16 years in workforce without job, percent blue‐collar workers, and median rent) developed by Yost were not available in China 24. Thus, GDP per capita, percentage of illiteracy aged 15 and over, per capita annual income of urban household, number of hospital beds per 1000 people, number of health technical personnel per 1000 people, healthy life expectancy, and the infant mortality rate (IMR) were included as a surrogate for area‐level SES. All of variables of eight districts or provinces were obtained from annual reports issued by National Bureau of Statistics and National Health and Family Planning Commission in China from 2005 to 2014. Then we used a comprehensive socioeconomic index to represent the SES of each area based on these variables.

### Statistical methods

A standardized index score was created to represent the area‐level SES by combining the seven indicator variables as defined above using principal component analysis (PCA). The number of components retained for extraction was based on the Kaiser criterion (eigenvalue ≥1.0). According to PCA result, the standardized component scores for the eight areas were sorted and ranked into tertiles (factor scores ranging from low [first tertile] to high [third tertile]).

Descriptive analysis was conducted tabulating the demographic and socioeconomic characteristics of the study population. Comparison of proportions between the stage and SES was made using the chi‐square test or Fisher's exact test. Multivariate analysis was undertaken using binary logistic regressions to describe association between individual/area‐level SES and stage at diagnosis after adjusting for age, sex, marital status, body mass index (BMI), medical insurance type, smoking, drinking, and history of respiratory diseases. In addition, we considered analysis of categorical data using a multinomial logit model to explore the effect across tertiles on stage among lung cancer patients. The statistical analyses were performed using SAS version 9.4 (SAS Institute Inc, Cary, NC).

## Results

A total of 5262 men and 1922 women diagnosed with incident lung cancer between 2005 and 2014 were collected from seven regions in China. Table 1 shows the seven census indicator variables of SES and the standardized index score for eight provinces in these regions. In general, all the indicator variables (except life expectancy and IMR) were significantly different among the areas (*P *< 0.001). Yunnan, Gansu, and Anhui provinces were assigned the lowest tertile group, Guangxi and Hunan provinces were the second tertile group. Shanxi, Liaoning and Zhejiang provinces were ranked as the highest tertile group with highest levels in economic development, medical resources allocation, and health care quality.

**Table 1:** PCA score created from seven census indicator variables of area‐level SES for the seven regions, 2005–2014

Characteristics of eight provinces in seven regions	Census indicator variables of area‐level SES							PCA score^h^
	GDP per capital^a^	Illiteracy percentage^b^	Household income^c^	HB number^d^	HP number^e^	Life expectancy^f^	IMR^g^	
Yunnan (Southwest)	16,303 ± 6840	11.95 ± 4.64	15,122.84 ± 4629.08	3.33 ± 0.91	3.30 ± 0.60	69.54	65.8	−1.843
Gansu (Northwest)	15,997 ± 6735	13.93 ± 5.85	12,640.25 ± 3514.97	3.29 ± 0.92	3.74 ± 0.59	72.23	31.5	−1.153
Anhui (East)	20,311 ± 9354	11.99 ± 4.55	15,211.96 ± 4782.09	2.76 ± 0.87	3.18 ± 0.64	75.08	26.1	−0.623
Guangxi (South)	19,982 ± 8764	4.87 ± 1.74	15,700.66 ± 4593.49	2.72 ± 0.91	3.65 ± 0.94	75.11	44.0	−0.533
Hunan (Central)	23,993 ± 10,683	4.87 ± 1.80	15,899.19 ± 4541.84	3.30 ± 1.13	3.82 ± 0.68	74.70	38.1	−0.229
Shanxi (North)	24,663 ± 8729	3.49 ± 1.18	15,079.93 ± 4415.41	3.94 ± 0.71	5.13 ± 0.59	74.92	19.2	0.536
Liaoning (Northeast)	40,964 ± 16,900	2.94 ± 1.07	17,173.21 ± 5725.96	4.58 ± 0.76	5.42 ± 0.34	76.38	18.7	1.493
Zhejiang (East)	49,964 ± 15,673	7.79 ± 2.47	26,585.88 ± 7281.08	3.56 ± 0.56	5.73 ± 0.97	77.73	17.1	2.352

a Yuan per person per year (mean ± SD, 2005–2014).

b Percentage of illiteracy aged 15 and over (%) (mean ± SD, 2005–2014).

c Annual income per capita of urban household (yuan per person per year) (mean ± SD, 2005–2014).

d Number of hospital beds per 1000 people (mean ± SD, 2005–2014).

e Number of health technical personnel per 1000 people (mean ± SD, 2005–2014).

f Life expectancy (years) only for 2010 (mean).

g Infant mortality rate (‰) only for 2010 (mean).

h Following the principle component analysis, the two factors extracted from the variables were retained with eigenvalue ≥ 1.0, which can explain 79.9% of the variance.

Overall 7013 cases were staged with a detailed assessment of the tumor stage, while fewer cases (2.4%) were presented as unknown or not applicable stage. As can be seen in Table 2, patients with advanced‐stage (IIIB‐IV) lung cancer accounted for 42.5% of the total population. Patients older than 75 years were less likely than those younger than 50 years to have advanced‐stage lung cancer (39.5% vs. 53.4%, *P *< 0.001). We also observed an obvious difference in stage distribution for people with BMI ≥ 30 and BMI < 25 groups (24.7% vs. 41.7%, *P *< 0.001). The proportion of current and ever‐smokers at diagnosis was 57.0%. In the individual‐level SES analysis, the educational level was slightly negatively associated with lung cancer stage. The proportion of patients with advanced stage in the illiterate group (47.7%) was higher compared to the primary school (44.7%), middle school (44.8%), high school (41.9%), and college degree or above group (46.7%). Farmers were more likely to be diagnosed at advanced stage than nonfarmers (46.2% vs. 42.8%, *P *< 0.05). Although no negative SES gradient persisted in the tertile group, we found a massive decrease in percentage of 22.4% for advanced‐stage cases in the highest tertile group compared with cases in the lowest tertile group (25.5% vs. 47.9%, *P *< 0.001).

**Table 2:** Demographic and socioeconomic index of lung cancer cases by stage at diagnosis, 2005–2014

Characteristic	Nonadvanced stage^a^	Advanced stage^b^	Unstaged	*χ* b, c
	No. (%)	No. (%)	No. (%)	*P*
Sex				
Male	2909 (55.3)	2210 (42.0)	143 (2.7)	0.262
Female	1048 (54.5)	846 (44.0)	28 (1.5)	
Age at diagnosis, years				
<50	219 (42.2)	277 (53.4)	23 (4.4)	<0.001
50–74	2870 (55.4)	2192 (42.3)	118 (2.3)	
≥75	868 (58.5)	587 (39.5)	30 (2.0)	
Marital status				
Single	41 (62.1)	23 (34.9)	2 (3.0)	0.343
Married	3874 (55.0)	3005 (42.6)	169 (2.4)	
Widowed/divorced	22 (62.9)	13 (37.1)	0 (0.0)	
Missing	20 (57.1)	15 (42.9)	0 (0.0)	
Body mass index				
<25	2913 (56.2)	2160 (41.7)	111 (2.1)	<0.001
25–30	679 (63.1)	369 (34.3)	29 (2.7)	
≥30	54 (70.1)	19 (24.7)	4 (5.2)	
Missing	311 (36.8)	508 (60.1)	27 (3.2)	
Medical insurance type				
UBMI	1209 (57.2)	855 (40.5)	48 (2.3)	<0.001
NRCMI	996 (53.2)	848 (45.3)	30 (1.6)	
Commercial insurance	101 (82.8)	21 (17.2)	0 (0.0)	
Social medicine	39 (39.0)	59 (59.0)	2 (2.0)	
Self pay	994 (57.7)	682 (39.6)	46 (2.7)	
Others	40 (78.4)	9 (17.7)	2 (3.9)	
Missing	578 (48.1)	582 (48.4)	43 (3.6)	
Smoking				
Current smoker	1820 (58.7)	1197 (38.6)	82 (2.7)	<0.001
Ex‐smoker	544 (54.8)	423 (42.6)	25 (2.5)	
Never smoker	1539 (51.2)	1409 (46.8)	61 (2.0)	
Missing	54 (64.3)	27 (32.1)	3 (3.6)	
Drinking				
Yes	1110 (54.4)	882 (43.2)	50 (2.5)	0.586
No	2575 (53.7)	2107 (43.9)	116 (2.4)	
Missing	272 (79.1)	67 (19.5)	5 (1.5)	
History of respiratory diseases				
Yes	286 (61.4)	167 (35.8)	13 (2.8)	<0.05
No	3671 (54.6)	2889 (43.0)	158 (2.4)	
Educational level				
Illiterate	397 (49.1)	386 (47.7)	26 (3.2)	0.198
Primary school	605 (52.8)	513 (44.7)	29 (2.5)	
Middle school	662 (52.6)	563 (44.8)	33 (2.6)	
High school	419 (55.1)	319 (41.9)	23 (3.0)	
College degree or above	222 (51.6)	201 (46.7)	7 (1.6)	
Missing	1652 (59.5)	1074 (38.7)	53 (1.9)	
Occupational level				
Nonfarmer	1749 (54.8)	1366 (42.8)	74 (2.3)	<0.05
Farmer	1518 (51.3)	1367 (46.2)	76 (2.6)	
Missing	690 (66.7)	323 (31.2)	21 (2.0)	
Area SES tertile				
1 (lowest)	1268 (46.1)	1317 (47.9)	167 (6.1)	<0.001
2	848 (43.2)	1110 (56.6)	4 (0.2)	
3 (highest)	1841 (74.5)	629 (25.5)	0 (0.0)	

UBMI, urban basic medical insurance; NRCMI, new rural cooperative medical insurance.

a Nonadvanced stage: StageI–IIIA.

b Advanced stage: Stage IIIB–IV.

c Participants who were diagnosed with unstaged lung cancer or lack of demographic information were not included in chi‐square test or the Fisher exact test. And none of the variables meet the criteria of Fisher exact test.

To further explore the association between demographic characteristics, individual/area‐level SES, and having advanced‐stage (IIIB‐IV) disease at diagnosis, we used binary logistic regression and implemented stratified analysis by sex (Table 3). In the unadjusted models, the OR for having advanced‐stage lung cancer among patients with the least versus most deprived tertile of area‐level SES was 0.35 (95%CI 0.30–0.40) for men, 0.29 (95%CI 0.23–0.36) for women, and 0.33 (95%CI 0.29–0.37) for both. Adding demographic variables attenuated the OR to 0.37 (95%CI 0.32–0.44) for men, 0.39 (95%CI 0.30–0.50) for women, and 0.37 (95%CI 0.33–0.42) in both. On the contrary, an inverse relationship was observed for middle versus most deprived tertile of area‐level SES. Nonfarmers (e.g., government employee, company employee, self‐employed Individuals, manual worker) for women and the both sexes were less likely to be diagnosed at advanced stage than farmers before adjustment for age, BMI and other factors (OR = 0.75; 95%CI 0.62–0.92 in women; OR = 0.87; 95%CI 0.78–0.96 in both sexes). After controlling for all demographic variables, a negative statistically significant association remained between educational SES and having advanced‐stage lung cancer for all subgroups of patients except high school and college degree or above in women.

**Table 3:** Odds ratios for presenting with advanced‐stage (IIIB‐IV) lung cancer by individual‐ and area‐level SES indices and demographic variables stratified by sex, 2005–2014, *n* = 7013

	Male				Female				Both			
	OR (95% CI)^a^	*P*	Adjusted OR	*P*	OR (95% CI)^a^	*P*	Adjusted OR	*P*	OR (95% CI)^a^	Adjusted OR	*P*
			(95% CI)^b^				(95% CI)^c^			(95% CI)^d^		
Age at diagnosis, years												
<50	1.00		1.00		1.00		1.00		1.00		1.00	
50–74	0.66 (0.53–0.84)	<0.001	0.70 (0.55–0.90)	0.006	0.53 (0.39–0.72)	<0.001	0.67 (0.49–0.91)	0.001	0.60 (0.50–0.73)	<0.001	0.67 (0.55–0.81)	<0.001
≥75	0.62 (0.48–0.80)	<0.001	0.67 (0.51–0.88)	0.004	0.40 (0.28–0.57)	<0.001	0.58 (0.39–0.86)	<0.001	0.54 (0.44–0.66)	<0.001	0.62 (0.50–0.78)	<0.001
Marital status												
Single	1.00				1.00				1.00			
Widowed/Divorced	1.15 (0.42–3.15)	0.785			0.88 (0.16–4.87)	0.879			1.05 (0.45–2.48)	0.905		
Married	1.37 (0.78–2.40)	0.278			1.42 (0.41–4.86)	0.580			1.38 (0.83–2.31)	0.216		
Body mass index												
<25	1.00		1.00		1.00		1.00		1.00		1.00	
25–30	0.73 (0.62–0.87)	<0.001	0.77 (0.65–0.92)	0.004	0.73 (0.56–0.94)	0.015	0.80 (0.61–1.05)	0.120	0.73 (0.64–0.84)	<0.001	0.78 (0.68–0.91)	0.001
≥30	0.25 (0.11–0.56)	<0.001	0.31 (0.13–0.71)	0.006	0.99 (0.47–2.11)	0.979	1.12 (0.50–2.51)	0.787	0.48 (0.28–0.80)	0.006	0.56 (0.32–0.98)	0.043
Medical insurance type												
UBMI	1.00		1.00		1.00		1.00		1.00		1.00	
NRCMI	1.14 (0.99–1.33)	0.076	0.97 (0.80–1.17)	0.731	1.37 (1.07–1.75)	0.012	1.06 (0.77–1.44)	0.786	1.20 (1.06–1.37)	0.004	0.99 (0.84–1.16)	0.829
Commercial insurance	0.29 (0.17–0.50)	<0.001	0.65 (0.37–1.16)	0.139	0.32 (0.12–0.84)	0.022	0.77 (0.28–2.17)	0.655	0.29 (0.18–0.47)	<0.001	0.69 (0.42–1.14)	0.144
Social medicine	2.04 (1.29–3.21)	0.002	1.34 (0.84–2.14)	0.232	2.66 (0.97–7.31)	0.058	1.64 (0.57–4.69)	0.379	2.14 (1.41–3.24)	<0.001	1.38 (0.90–2.12)	0.142
Self pay	0.98 (0.84–1.14)	0.759	0.86 (0.73–1.02)	0.081	0.96 (0.74–1.24)	0.750	0.78 (0.59–1.04)	0.080	0.97 (0.85–1.11)	0.651	0.83 (0.72–0.96)	0.012
Others	0.31 (0.13–0.76)	0.010	0.36 (0.14–0.92)	0.032	0.34 (0.09–1.19)	0.091	0.54 (0.14–2.04)	0.377	0.32 (0.15–0.66)	0.002	0.40 (0.19–0.86)	0.018
Smoking												
Never smoker	1.00		1.00		1.00		1.00		1.00		1.00	
Ex‐smoker	0.76 (0.64–0.90)	0.002	1.05 (0.87–1.27)	0.678	0.65 (0.31–1.37)	0.257	1.21 (0.53–2.74)	0.650	0.85 (0.73–0.98)	0.029	1.13 (0.96–1.32)	0.145
Current smoker	0.65 (0.56–0.74)	<0.001	0.69 (0.60–0.80)	<0.001	0.37 (0.21–0.65)	<0.001	0.83(0.45–1.52)	0.544	0.72 (0.65–0.80)	<0.001	0.74 (0.66–0.82)	<0.001
Drinking												
Yes	1.00				1.00				1.00			
No	1.00 (0.89–1.12)	0.969			1.49 (0.85–2.59)	0.164			1.03 (0.93–1.14)	0.586		
History of respiratory diseases												
Yes	1.00				1.00		1.00		1.00		1.00	
No	1.18 (0.95–1.46)	0.138			2.70 (1.58–4.61)	<0.001	2.50 (1.39–4.48)	0.002	1.35 (1.11–1.64)	0.003	1.40 (1.14–1.73)	0.002
Educational level												
illiterate	1.00		1.00		1.00		1.00		1.00		1.00	
Primary school	0.95 (0.76–1.18)	0.624	0.72 (0.57–0.91)	0.006	0.78 (0.57–1.08)	0.136	0.62 (0.44–0.88)	0.006	0.87 (0.73–1.05)	0.143	0.69 (0.57–0.84)	<0.001
Middle school	0.97 (0.78–1.20)	0.776	0.70 (0.55–0.89)	0.003	0.75 (0.53–1.05)	0.089	0.59 (0.40–0.85)	0.004	0.88 (0.73–1.05)	0.144	0.68 (0.56–0.82)	<0.001
High school	0.82 (0.64–1.04)	0.098	0.56 (0.43–0.73)	<0.001	0.87 (0.58–1.30)	0.483	0.74 (0.47–1.16)	0.166	0.78 (0.64–0.96)	0.018	0.59 (0.47–0.74)	<0.001
College degree or above	0.99 (0.75–1.31)	0.951	0.58 (0.42–0.79)	<0.001	0.91 (0.57–1.45)	0.684	0.63 (0.37–1.07)	0.064	0.93 (0.74–1.18)	0.555	0.60 (0.46–0.79)	<0.001
Occupational level												
Farmer	1.00		1.00		1.00		1.00		1.00		1.00	
Nonfarmer	0.92 (0.82–1.03)	0.156	0.97 (0.83–1.14)	0.716	0.75 (0.62–0.92)	0.005	0.84 (0.65–1.09)	0.201	0.87 (0.78–0.96)	0.006	0.94 (0.82–1.07)	0.362
Area SES tertile												
1 (lowest)	1.00		1.00		1.00		1.00		1.00		1.00	
2	1.30 (1.13–1.49)	<0.001	1.53 (1.32–1.78)	<0.001	1.18 (0.93–1.49)	0.180	1.67 (1.28–2.20)	<0.001	1.26 (1.12–1.42)	<0.001	1.57 (1.38–1.79)	<0.001
3 (highest)	0.35 (0.30–0.40)	<0.001	0.37 (0.32–0.44)	<0.001	0.29 (0.23–0.36)	<0.001	0.39 (0.30–0.50)	<0.001	0.33 (0.29–0.37)	<0.001	0.37 (0.33–0.42)	<0.001

OR, odd ratio; CI, confidence interval.

a Result of univariate methods before multiple logistic regression analysis.

b Adjusted for age, BMI, medical insurance type, smoking, educational level, occupational level, and area‐level SES of men in binary logistic regression model.

c Adjusted for age, BMI, medical insurance type, smoking, history of respiratory diseases, educational level, occupational level, and area‐level SES of women in binary logistic regression model.

d Adjusted for age, BMI, medical insurance type, smoking, history of respiratory diseases, educational level, occupational level, and area‐level SES of the whole population in binary logistic regression model.

Table 4 summarizes multinomial logit model results for the association between stage and individual/area‐level SES when all the other demographic factors are considered. Model 1 was the baseline model including education, occupation, and area‐level SES characteristics. Model 2 (including demographics variables) slightly attenuated the individual‐ and area‐level socioeconomic effects.

**Table 4:** Odds ratios for presenting with higher stage lung cancer by socioeconomic status in multinomial logit models, 2005–2014, *n* = 7013

		Model 1^a^						Model 2^b^					
		Stage IV		Stage III		Stage II		Stage IV		Stage III		Stage II	
Socioeconomic	status	OR (95% CI)	*P*	OR (95% CI)	*P*	OR (95% CI)	*P*	OR (95% CI)	*P*	OR (95% CI)	*P*	OR (95% CI)	*P*
Educational level	Illiterate	1.00		1.00		1.00		1.00		1.00		1.00	
	Primary school	0.61 (0.46–0.81)	<0.001	0.89 (0.68–1.16)	0.381	1.32 (0.95–1.84)	0.102	0.64 (0.48–0.85)	0.002	0.86 (0.66–1.13)	0.287	1.27 (0.91–1.78)	0.154
	Middle school	0.69 (0.52–0.91)	0.009	0.94 (0.71–1.23)	0.625	1.45 (1.04–2.01)	0.030	0.71 (0.53–0.94)	0.016	0.89 (0.68–1.17)	0.403	1.38 (0.99–1.93)	0.059
	High school	0.58 (0.42–0.80)	0.001	0.87 (0.64–1.18)	0.364	1.61 (1.12–2.33)	0.011	0.63 (0.46–0.88)	0.006	0.84 (0.61–1.15)	0.265	1.56 (1.07–2.26)	0.020
	College degree or above	0.55 (0.38–0.79)	0.001	0.67 (0.47–0.96)	0.029	1.02 (0.65–1.58)	0.942	0.52 (0.36–0.76)	<0.001	0.65 (0.45–0.93)	0.019	1.02 (0.65–1.60)	0.943
Occupational level	Farmer	1.00		1.00		1.00		1.00		1.00		1.00	
	Nonfarmer	0.75 (0.63–0.88)	<0.001	0.71 (0.60–0.83)	<0.001	0.77 (0.64–0.93)	0.006	0.88 (0.72–1.06)	0.180	0.76 (0.63–0.91)	0.003	0.85 (0.69–1.06)	0.144
Area SES tertile	1 (lowest)	1.00		1.00		1.00		1.00		1.00		1.00	
	2	1.22 (1.01–1.47)	0.042	1.32 (1.09–1.59)	0.004	0.80 (0.64–1.01)	0.060	1.41 (1.16–1.72)	<0.001	1.39 (1.14–1.69)	0.001	0.80 (0.63–1.01)	0.062
	3 (highest)	0.23 (0.20–0.28)	<0.001	0.60 (0.51–0.70)	<0.001	0.75 (0.62–0.90)	0.002	0.29 (0.24–0.35)	<0.001	0.67 (0.56–0.79)	<0.001	0.77 (0.63–0.94)	0.012

a Model 1: multivariate analysis: education/occupation/area‐level SES. Stage I is the reference group.

b Model 2: multivariate analysis: education/occupation/area‐level SES+all demographics index after a stepwise selection. Stage + is the reference group.

Those who had received higher education, and were living in the highest tertile of area‐level SES appeared to have decreased risk of advanced‐stage lung cancer, when controlled for individual SES and all demographics index (model 2). Especially patients who belonged to least deprived tertile of area‐level SES experienced lower advance‐stage lung cancer risks (OR_stage II_ = 0.77[95%CI =0.63–0.94]); OR_stageIII_ = 0.67[95%CI = 0.56–0.79]; OR_stage IV_ = 0.29[95%CI = 0.24–0.35]). However, patients who belonged to the median tertile of area‐level SES had a 39% increased risk of having stage III (OR = 1.39[95%CI =1.14–1.69]), 41% increased risk of having stage IV (OR = 1.41[95%CI = 1.16–1.72]) compared with cases of the lowest tertile. Educational SES index was also associated with the stage IV disease. Patients with higher education were 36%, 29%, 37%, and 48% less likely to having stage IV lung cancer, compared with the illiterate subgroup (OR = 0.64[95%CI = 0.48–0.85] for primary school; OR = 0.71[95%CI = 0.53–0.94] for middle school; OR = 0.63[95%CI = 0.46–0.88] for high school; OR = 0.52[95%CI = 0.36–0.76] for college degree or above. Nonfarmers seemed to have a less risk of having higher stages at diagnosis compared with farmers before the adjustment, but the staging differences across occupation had shown no significant effects when the demographic factors were added to the model.

## Discussion

This study showed that a clinical relevance existed between socioeconomic disparities and stage at diagnosis for primary lung cancer in mainland China. In this hospital‐based analysis from eight provinces, the least socioeconomically deprived areas were associated with a lower risk of having advanced‐stage lung cancer, whereas patients who belong to the median deprived areas had a significant increased risk of having advanced‐stage cancer when compared with patients from the most deprived areas. Moreover, we also found increasing educational level was associated with a decreased risk to be diagnosed at advanced‐stage lung cancer in both men and women. To the best of our knowledge, this is the first study to consider, simultaneously, measures of socioeconomic status at the individual and regional levels and their influence on primary lung cancer clinical outcomes in mainland China.

Information on SES for individuals is not reported in the current national cancer registry database 25. Therefore, following the recommendation regarding measures of social class for public health research and surveillance by Krieger, we used the occupational and educational categories as a proxy measure of individual SES, with the consideration of unavailable income information in our CRF database. For the occupation category, the best known and longest employed of the occupational class measure is the British Registrar General's social class schema which is based on skill and status. In the USA, the census occupational data can be meaningfully grouped to create a class‐based measure (e.g., the administrative support, sales, and other six census‐defined occupational groups are defined as working class). The occupation data collected from the medical records of the study is different from the USA. There are eight occupational groups including government worker, corporate personnel, office staff, self‐employed individuals, freelancer, soldier, unemployed, and farmer/rural migrant worker. Obviously, the occupational class from the records can not reflect the difference between material and social resources and assets. However, Farmers or rural migrant workers are a huge and special group in China and their identity is different from citizen and other occupational groups. As a vulnerable group in China, the farmers are poorly educated and skilled, with limited access to health care. So the classification in our study can capture occupations as a measure of what Stevenson termed “standing within the community” or “culture” 23.

We found that patients with higher education degree incurred a moderate decrease at the advanced stage at diagnosis. In fact, many studies revealed a similar association between education/income and cancer outcome and the impact varied by race/ethnic, smoking, insurance style, and cancer screening program 19, 26, 27, 28, 29, 30. Moreover, the two studies suggested that more deprived patients were likely to present with more advanced‐stage cancer in non‐UHCS in the USA. One study from Silverstein et al. suggested that lower socio‐economic position (using per capital, income and education as measure of SEP) were likely to present with distant stage at diagnosis compare to high SEP without statistical significance (OR = 1.06[95%CI = 0.28‐3.96]). 31. The other study from Schwartz et al. used the aggregate SES variable (including occupation, poverty, education, and age) found to contribute significantly to risk of nonlocalized stage in higher compared to low SES (OR = 1.28[95%CI =1.12–1.45]) 20. The most other studies found no association according to a recent systematic review 14. There were several plausible explanatory factors for this phenomenon in China. However, individuals with low educational level or low income seem to be less likely to seek medical advice or undergo treatment for a cough or hoarseness, prone to smoking 32, or lived close to cancer causing substances, such as asbestos, arsenic, coal, and diesel engine exhaust 9, 33. According to the result of the 2015 China Adult Tobacco Survey, the percent of current smoking rate with low education level (middle school degree or below) was higher (60.0%) compared with high education level (college degree or above) in men (41.9%) 34. Friedemann et al., reported being a smoker was associated with reduced likelihood of help‐seeking, and one contributor to late‐stage diagnosis could be patient delay in help‐seeking 35.Moreover, public hospitals and medical centers that can provide routine physical examination or the lung cancer screening program were primarily located in the advantaged district of urban areas, and the medical expenditure for diagnosis and treatment seemed catastrophic for low‐income patients with lung cancer in China.

For the area‐level or neighborhood SES, the illiteracy percentage index, the household income index, and other typical indices are frequently used in order to judge area‐level SES in most studies. However, there are no certain criteria of judging the degree of area‐level social‐economic status. Each researcher utilized these SES indices according to their own criteria so far 9, 36, 37. So there is lack of comparative data for the several indicators. In this situation, we used a multifactorial socioeconomic index which was created from census indicator variables of SES (education index, median household income) developed by Yost and some new variables (GDP per capita, number of hospital beds per 1000 people, number of health technical personnel per 1000 people, healthy life expectancy, and the infant mortality rate). Each variable measures a different aspect of area‐level SES, capturing area‐level SES more aptly. Considering strong correlation between the selected input variables, the PCA technique effectively deals with multicollinearity. The deprivation indices represented an attempt to more accurately reflect the multidimensional character of regional socioeconomic position. We identified low area‐level SES is an independent indication in diagnosing advanced‐stage lung cancer after adjusting for demographic variables. However, we did not observe dose–response gradients between area‐level SES and stage. The negative association was entirely limited to the most versus least deprived tertile of area‐level SES, suggesting that some confounding factors could influence cancer staging. Hystad et al. found no linear dose–‐response relationships were observed for unadjusted or adjusted models and the elevated ORs of 1.66 were only restricted to the lowest quintile of neighborhood SES index 29. Moreover, we also found some conflicting result about relationship between the median deprived areas index and stage. Our data showed that an observed increasing probability to be diagnosed at stage IIIB or IV in median versus most deprived areas after adjusting for demographic variables (ORs, 1.39, 1.41). Given that some unmeasured risk factors such as individual health‐related behaviors and access to health may contribute to the development and progression of lung cancer, it is hypothesized that, with the improvement of living conditions, individuals living in the median deprived areas may be more likely to eat energy‐dense, nutrient‐poor foods, prone to smoking more often, and adopt sedentary behaviors than those of lowest area‐level SES 38, 39. Meanwhile they still had worse access to health services than those with highest area‐level SES. And because the eight provinces covered large geographic areas and tens of millions of people, the area‐level SES measures may not capture different exposure factors. More research is needed to examine the association between SES and lung cancer in a smaller unit, such as community level considering other important etiological factors.

Our study has some potential limitations that should be considered in the interpretation of the results. First, we did not include some indicator variables of SES, such as annual personal income, percent of older than 16 years in workforce without job in our analysis, which may fail to capture some implication for outcome. We used annual average of census data in 2005–2014 to minimize error in determining relevant time period to estimate area‐level SES. Second, we used the hospital‐based data and the patients from the leading public cancer hospital of the province may not represent the whole population of the area. Those who chose the highest level hospital, in other words, the most expensive hospital may be different from those who chose township‐ or municipal‐level hospitals. As a result, the demographic and socioeconomic characteristic of the cancer cases from the eight hospitals may be different from the common people nationwide. Third, considering relevant pathways by which socioeconomic status may affect the stage of lung cancer, our analyses could benefit from the addition of other useful information such as working circumstance, the effect of air pollution and cancer screening program in the targeted area. Fourth, we used the convenience sampling instead of random sampling methods. We give the general framework for sampling considering the number of cases in each month, that is to say, the clerks should extract one hundred medical reports in one month every year. Because there were less than one hundred cases in January and February in most years of most hospitals, we had to choose two or three months to extract the information if we included the two months. Thus we excluded January and February in the convenience sampling process. In addition, the method of this sampling has been used in a similar retrospective clinical epidemiological study of breast cancer, which has published more than 20 papers 40.

In conclusion, our study outlines a reproducible approach to the development of SES indices at the individual and area levels simultaneously according to readily available census data in China. Using data from seven socio‐demographically diverse regions, it shows that a clinical relevance existed between socioeconomic disparities and lung cancer stage at diagnosis in China. These results provide evidence that public health policy makers should allocate efficiently, the limited medical resource to those socially deprived individuals, such as farmers or rural migrant workers with low education background, and provide better accessibility to undertake diagnosing, health lifestyle‐related education information for rural and undeveloped areas.

## Conflicts of Interest

Authors report no financial disclosures or conflict of interest.
